# Perioperative management of anti–TIF1-γ dermatomyositis in a patient with breast cancer: a case report

**DOI:** 10.3389/fmed.2026.1876664

**Published:** 2026-07-08

**Authors:** YaQian Li, YuTing Wang, Gang Chen

**Affiliations:** Department of Anesthesiology, Sir Run Run Shaw Hospital, Zhejiang University School of Medicine, HangZhou, China

**Keywords:** anesthesia, dermatomyositis, myositis-specific autoantibodies, perioperative management, TIF1-γ

## Abstract

Dermatomyositis (DM) is a rare autoimmune disease, and its subtypes exhibit considerable heterogeneity in perioperative risk. The anti-transcription intermediary factor 1-gamma (TIF1-γ) antibody–positive subtype is particularly associated with an increased risk of underlying malignancy and poses unique anesthetic challenges because of its potential multisystem involvement. We report the perioperative anesthetic management of a 47-year-old woman with TIF1-γ antibody–positive DM complicated by breast cancer who underwent a modified radical mastectomy. The patient presented with classic cutaneous manifestations, proximal myopathy, and oropharyngeal dysphagia. Comprehensive pre-operative anesthetic assessment and meticulous perioperative management contributed to an uneventful surgical course and favorable outcome. This case highlights the marked clinical heterogeneity of DM subtypes and the corresponding differences in perioperative anesthetic risk. It also underscores the importance of recognizing the potential multisystem involvement of DM in patients undergoing anesthesia. Thorough pre-operative evaluation and individualized anesthetic management are therefore essential for optimizing perioperative outcomes.

## Introduction

Dermatomyositis (DM) is a systemic inflammatory disease characterized by chronic muscle inflammation. Extracutaneous manifestations include rash, interstitial lung disease, arthritis, and cardiac involvement (1). Additionally, patients with DM are in a hypercoagulable state, and the risk of pulmonary embolism is significantly higher than that of the general population (2). Given this wide spectrum of clinical presentations, perioperative anesthesia management in these patients can be challenging. Myositis-specific autoantibodies are closely associated with clinical phenotypes and serve as useful predictors of organ involvement.

The patient in this case report had TIF1-γ+ DM. In addition to rash and myositis, hallmark features of this subtype include a strong association with severe dysphagia (3–5) and malignancy (5–7). Both of which are particularly characteristic features. The major perioperative anesthesia challenges in such patients include comprehensive pre-operative risk assessment, prevention of aspiration and reflux, protection of respiratory function, and prevention and early recognition of pulmonary embolism. After multidisciplinary pre-operative consultation to optimize the patient's overall condition and the development of an individualized anesthetic plan, the patient had a smooth perioperative course.

## Case presentation

A 47-year-old female patient was admitted due to a marked elevation of creatine kinase (CK) during chemotherapy for breast cancer. Physical examination revealed red papules and pustules on the chest, upper arms, and back. The patient complained of muscle pain, decreased muscle strength, and dysphagia. Two days after admission, she developed further reduction in limb muscle strength, difficulty in lifting the neck flexors, and worsening dysphagia.

Multidisciplinary consultation involving the Departments of Dermatology, Otorhinolaryngology, Neurology, and Rheumatology and Immunology led to a clinical diagnosis of DM. Imaging revealed atrophy with extensive exudation changes in the bilateral lower limb muscles. Myositis-specific antibody testing was positive for anti-TIF1-γ antibody. Methylprednisolone was initiated. Given the close association between DM disease activity and tumor burden in this patient, surgical treatment of the breast cancer was planned once her clinical condition had stabilized.

After 6 days of treatment, the patient's muscle strength improved and CK levels declined substantially, making surgical intervention feasible. A comprehensive pre-operative anesthetic evaluation and risk assessment were subsequently performed.

### Pre-operative anesthesia evaluation

The patient had no significant past medical history, including chronic illnesses, autoimmune diseases, or a family history of malignancy. She has been diagnosed with TIF1-γ antibody-positive DM complicated by breast cancer. Given the potential multisystem involvement of DM, the following complications warrant particular attention: interstitial lung disease (ILD), muscle weakness, myocarditis, and hypercoagulability. Recommended workup includes pulmonary function tests, echocardiography, lower-extremity venous ultrasonography, repeat electrocardiography (ECG), myocardial enzyme panel, and D-dimer assay. In addition, dysphagia—a characteristic manifestation associated with TIF1-γ–and the consequent risk of malnutrition were also evaluated.

### Assessment findings

Pulmonary function tests were within normal limits. Echocardiography demonstrated mild-to-moderate aortic regurgitation. ECG revealed sinus rhythm (93 bpm) with mild T-wave abnormalities in the inferior, anterior, and lateral leads, unchanged from those observed on admission.

CK-MB was 5.76 ng/ml, representing a marked decrease from 20.57 ng/ml at admission. Lower extremity venous ultrasonography was negative for deep vein thrombosis. D-dimer was 3.87 μg/ml, compared with 3.42 μg/ml on admission, without a clinically significant increase.

Neurological examination demonstrated improvement in proximal limb and neck flexor muscle strength from Medical Research Council (MRC) grade 3 to grade 4. However, dysphagia showed minimal improvement. The patient currently presents with a productive cough and bilateral coarse breath sounds. Laryngoscopy revealed pharyngolaryngeal mucosal edema and hyperemia. The Functional Oral Intake Scale (FOIS) was rated 3–4, and the Water Swallowing Test (WST) was grade 4.

Nasogastric tube feeding was recommended by the nutrition team, but the patient and her family declined. Small-volume oral feeding with enteral nutrition was advised, although episodes of coughing and choking were observed during oral intake. The patient was deemed to be at high risk for reflux and aspiration. Pantoprazole was administered pre-operatively for gastric acid suppression. Body weight remained stable throughout hospitalization, and despite mild hypoalbuminemia, overall nutritional status was considered acceptable.

The major perioperative anesthetic considerations include aspiration prophylaxis, respiratory function preservation, cautious use of neuromuscular blocking agents (NMBAs), and monitoring for pulmonary embolism (PE).

### Intraoperative anesthesia management

Standard monitoring included ECG, invasive arterial pressure, pulse oximetry, bispectral index (BIS), and core temperature. The patient had fasted for 8 h before surgery. Before induction, oropharyngeal secretions were thoroughly suctioned, and the patient was positioned in the reverse Trendelenburg position. Rocuronium (0.5 mg/kg) was chosen due to the availability of a specific reversal agent (sugammadex). Induction agents included ciprofol (4 mg/kg) and sufentanil (0.5 μg/kg). Following rapid sequence induction (RSI), tracheal intubation was performed once adequate neuromuscular blockade had been achieved, and the airway was subsequently suctioned. Anesthesia was maintained using total intravenous anesthesia (TIVA) in conjunction with a lung-protective ventilation strategy. Sufentanil was administered intermittently, and additional rocuronium (0.1 mg/kg) was given when spontaneous breathing resumed.

### Emergence phase

During emergence from anesthesia, quantitative neuromuscular monitoring was used in conjunction with clinical signs of recovery to guide tracheal extubation. Twenty minutes after discontinuation of anesthetic agents (approximately 2.5 h after the final dose of rocuronium), the patient regained consciousness. At that time, the train-of-four (TOF) ratio was 56%, and tidal volume was approximately 3 ml/kg. Five minutes after administration of sugammadex (2 mg/kg), the TOF ratio increased to 90% and tidal volume reached 7 ml/kg. After thorough suctioning of oropharyngeal secretions and confirmation that the patient was fully awake and had adequate spontaneous ventilation, the trachea was extubated uneventfully. A complete timeline of the patient's clinical course is presented in Table 1.

**Table 1:** Timeline of the patient's clinical course.

Time point	Clincial events
Four months before admission	A 47-year-old female patient was diagnosed with invasive breast cancer. Pathology ER (–), PR (–), Her-2 (3+), Ki-67 (80%). The patient received five cycles of chemotherapy with the TCbHP regimen.
Before admission	The patient developed red papules and pustules 20 days before admission, and dysphagia 9 days before admission, but did not seek medical attention.
Day 1 (admission)	The patient was admitted due to a marked elevation of creatine kinase (CK). CK 3319U/L.
Day 3	The patient experienced further decline in limb muscle strength (3/5), difficulty in lifting the head due to neck flexor weakness, worsening dysphagia, generalized body pain, facial edema, and ptosis.
Day 9	The nutrition department was consulted and recommended nasogastric tube feeding, but patient refused. Dermatomyositis was diagnosed based on the multidisciplinary consultation. Imaging studies showed atrophy with extensive exudation in the bilateral lower limb muscles. Myositis-specific antibody testing was positive for anti-TIF1-γ antibody.
Day 15	The patient received intravenous methylprednisolone 60 mg/day. Muscle strength (4/5) and CK levels improved (437U/L), surgical management of the breast cancer was planned. A pre-operative consultation and risk assessment was performed by the department of anesthesiology.
Day 16	After completing the relevant examinations, the patient underwent modified radical mastectomy.
Day19	Muscle strength grade 4+ and FOIS is rated 5.
Day 21	The patient was discharged, with follow-up scheduled in the rheumatology clinic.

### Post-operative course

Following extubation, the patient remained dysphagic and required regular clearance of oropharyngeal secretions. She was transferred to the surgical ward with no post-operative signs of aspiration pneumonia or pulmonary infection. After 24 h of routine oxygen therapy, oxygen was weaned successfully with normal oxygenation maintained. By post-operative day 3, both muscle strength and swallowing function had improved compared with pre-operative baseline. Proximal muscle strength had improved to MRC grade 4+, and FOIS score increased to 5. The remainder of the post-operative course was uneventful. The patient was discharged on post-operative day 5 in stable condition and scheduled for outpatient follow-up in the rheumatology clinic.

## Discussion

This report describes the key considerations in the pre-operative anesthetic assessment and perioperative management of a patient with TIF1-γ-positive DM, and also outlines the anesthesia risks associated with other DM subtypes, to provide a broader perspective on the anesthetic challenges inherent to this rare and heterogeneous disease.

DM is a major subtype of idiopathic inflammatory myopathy (IIM), with an estimated incidence of 2.9–34 cases per 100,000 individuals (2). The disease comprises five distinct subtypes, each characterized by specific myositis-related autoantibodies that are closely associated with clinical phenotypes and, consequently, with anesthesia-related risks. In patients with anti-TIF1-γ-positive DM, the major perioperative anesthetic challenges include a strong association with malignancy, aspiration risk and malnutrition due to dysphagia, ILD, myocarditis, thromboembolism, a history of long-term corticosteroid use, muscle weakness, and the judicious use of NMBAs (2, 8).

The probability of malignancy in patients with anti-TIF1-γ antibody positivity is as high as 42.6% (5). Dermatomyositis can occur either before or after the detection of the tumor. Therefore, tumor screening is indicated once the diagnosis is confirmed. Severe dysphagia is another characteristic manifestation of this subtype. Pre-anesthetic evaluation should focus on the severity of dysphagia, the risk of reflux and aspiration, and possible associated nutritional disorders and pulmonary infection, combine with assessment tools such as the FOIS and the WTS (9, 10). Early involvement of a nutrition specialist is very important. In addition, appropriate precautions for the above-mentioned risks should be implemented during both anesthesia induction and the recovery phase (5, 11). In this case, the patient's dysphagia showed no significant improvement. Thanks to early dietary guidance, no serious pre-operative complications occurred. Adequate fasting, pre-operative administration of a proton pump inhibitor, RSI with reverse Trendelenburg position, and thorough suctioning of oral secretions before and after induction and extubation to avoid reflux and aspiration. Intraoperative blood gas monitoring was conducted to maintain homeostasis.

Previous studies have shown that approximately 41% of patients with polymyositis /DM develop ILD (12). Myocardial inflammation can lead to left ventricular dysfunction and arrhythmias, which is one of the leading causes of death in DM (13). The risk of PE within the first year of polymyositis/DM is 16.44 times higher than in the general population (14). Screening for these high-incidence, serious complications is mandatory before anesthesia. Notably, muscle vascular injury due to inflammation may pre-dispose to anticoagulation-induced muscle hemorrhage (15). In this patient, D-dimer and lower extremity venous ultrasonography showed no significant abnormalities, and anticoagulation was therefore not initiated. However, if significant vital sign fluctuations occur during the perioperative period, the anesthesiologist should consider the possibility of PE.

The administration of NMBAs in patients with DM requires great caution. Even in patients with well-controlled disease, perifascicular vascular injury may alter NMBA pharmacodynamics, leading to prolonged duration of action and delayed reversal (16), intraoperative neuromuscular monitoring can be used to guide the administration of NMBAs. Of note, DM pre-dominantly affects proximal muscles, neuromuscular monitoring or grip strength may not fully represent the recovery of overall muscle strength, other indicators should be taken into consideration when extubation. Both balanced anesthesia or TIVA can be used safely in these patients. Lung-protective ventilation should be used intraoperatively to minimize respiratory impact. In this patient, TIVA was chosen mainly due to the potentiating effect of inhaled anesthetics on neuromuscular blockade.

Anesthesia management risks that are specific to other subtypes. anti-melanoma differentiation-associated gene 5 (MDA5) is linked to rapidly progressive interstitial lung disease, particularly in Asian populations. In this subtupy, perioperative oxygenation should be rigorously monitored to prevent adverse outcomes associated with the progression of interstitial pneumonia (17). Anti-nuclear matrix protein (NXP2) is associated with significant myositis and an increased risk of calcinosis. In this subtype, especially for patients with a prolonged disease course, perioperative management should account for the effects of calcinosis. Common challenges include difficulty with intubation and vascular access due to osteoarticular involvement. Although rare, serious sequelae may include reflux, hemorrhage, and perforation from gastrointestinal deposition, as well as life-threatening arrhythmias resulting from myocardial deposition (18). In patients with anti-small ubiquitin-like modifier activating enzyme (SMA) DM, typically cutaneous manifestations precede muscle involvement, and dysphagia is common. Because myositis symptoms are often subtle, this subtype is frequently underdiagnosed. Accordingly, perioperative vigilance is warranted in patients who present with characteristic rash and dysphagia (19). Anti-Mi-2 antibodies are associated with prominent myositis and elevated creatine kinase levels, whereas ILD is rare. This subtype predicts a good treatment response and favorable prognosis (20).

## Conclusion

This case highlights the importance of careful pre-operative anesthesia evaluation in patients with DM. Recognition of myositis-specific autoantibody subtypes may provide additional information that helps guide anesthetic planning and perioperative anesthesia risk management.

## Data Availability

The original contributions presented in the study are included in the article/supplementary material, further inquiries can be directed to the corresponding author.
